# Relative Risk of Cardiovascular Mortality in Breast Cancer Patients: A Population-Based Study

**DOI:** 10.31083/j.rcm2304120

**Published:** 2022-04-01

**Authors:** Chengshi Wang, Tao He, Zhu Wang, Dan Zheng, Chaoyong Shen

**Affiliations:** ^1^Department of Breast Surgery, Sichuan Cancer Hospital & Institute, Sichuan Cancer Center, School of Medicine, University of Electronic Science and Technology of China, 610044 Chengdu, Sichuan, China; ^2^Laboratory of Molecular Diagnosis of Cancer, Clinical Research Center for Breast, West China Hospital, Sichuan University, 610041 Chengdu, Sichuan, China; ^3^Department of Breast Surgery, West China School of Medicine/West China Hospital, Sichuan University, 610041 Chengdu, Sichuan, China; ^4^Department of Head, Neck and Mammary Gland Oncology, Cancer Center, West China Hospital, Sichuan University, 610041 Chengdu, Sichuan, China; ^5^Department of Gastrointestinal Surgery, West China Hospital, Sichuan University, 610041 Chengdu, Sichuan, China

**Keywords:** breast cancer, cardiovascular disease, mortality, population

## Abstract

**Aims:**

To investigate the risk of cardiovascular disease (CVD) mortality in breast cancer patients compared with the general female population.

**Methods:**

Data was retrieved from the Surveillance, Epidemiology, and End Results database. 924,439 female breast cancer patients who were at the age of follow-up ≥30 years and diagnosed during 1990–2016 as well as the aggregated general female population in the US were included. Using multivariable Poisson regression, we calculated incidence rate ratios (IRRs) of CVD mortality among female breast cancer patients compared with the female population.

**Results:**

The risk of CVD mortality was mildly increased among breast cancer patients at the age of follow-up 30–64 years (IRR 1.06, 95% confidence interval [CI] 1.03–1.10) compared with the general population. This growth of risk reached its peak within the first month after diagnosis (IRR 3.33, 95% CI 2.84–3.91) and was mainly activated by diseases of the heart (IRR 1.11, 95% CI 1.07–1.15). The elevation was greatest in survivors at the age of follow up 30–34 years (IRR 3.50, 95% CI 1.75–7.01).

**Conclusions:**

Clinicians should provide risk mitigation strategies with early monitoring of CVD mortality for breast cancer survivors, especially those who were young or with aggressive tumor stage.

## 1. Introduction

The incidence of breast cancer has been growing obviously over the past few decades, because of the improved survival rates with the development of cancer screening, diagnosis and treatments [1]. In the United States, there are nearly 300 thousand female breast cancer survivors in 2021, which is accounted for the largest number of newly diagnosed cancer [1].

The number of death from cardiovascular disease (CVD), which is considered as one of the leading causes of non-cancer death, is accounted for approximately 11.7% of breast cancer patients [2, 3], given that the risk of CVD death may be increased by cardiotoxicity of irradiation and chemotherapy as well as risk factors shared by CVD and patients with breast cancer [2, 4]. Previous studies have reported the increased absolute risk of CVD death in breast cancer patients (e.g., older age, receiving anthracycline, trastuzumab as well as left side radiotherapy) [2, 5]. However, the relative risk of CVD death among breast cancer survivors is controversial. Some studies suggested that from the point of cancer diagnosis forward into survivorship, breast cancer patients are at an increased hazard ratio of CVD-related mortality [3, 6], but this increase in risk is manifest approximately seven years after diagnosis [7] compared against the general population. Though, another study reported that the elevated risk of CVD mortality comes to its peak during the first week after breast cancer diagnosis [8]. In addition, other studies came to contrary conclusions. The risk of heart-specific mortality of breast cancer patients treated with radiotherapy or chemotherapy was lower compared with the general population [9]. Women with 70–79 years old at diagnosis of localized breast cancer had a lower risk of CVD mortality, compared to age-matched women without breast cancer [10]. The risk of cardiovascular mortality in heart failure patients did not differ by breast cancer status [11]. However, no studies so far have conducted a systematic conclusion on the relative risk of CVD mortality in breast cancer patients by simultaneously stratifying age at follow-up and time since cancer diagnosis relative to the general population.

Using the Surveillance, Epidemiology, and End Results database (SEER), we built up a large population-based cohort study covering females who were diagnosed with the first primary breast cancer and the corresponding female general population to assess the risk of CVD death. We focused on the impact of age at follow-up and time since diagnosis on the risk of death from CVD.

## 2. Patients and Methods

We performed a cohort study of female breast cancer survivors at the age of follow-up ≥30 years who were diagnosed from January 1, 1990 to December 31, 2016 in SEER. The SEER contains information on demographic, clinical characteristics, as well as follow-up from nine registries in 1973, expanding to 13 registries in 1992 and 18 in 2000, accounting for 28% of the U.S. population.

Because only the aggregated data was accessible, we included 7,161,749 person-years from the general female population during 1990–2016 from the United States. Using SEER, we still selected 1,189,576 breast cancer patients who were diagnosed as first primary by pathological identification from 1975 to 2016. Breast cancer patients and the general population in this analysis came from 13 states in America (the Northeast: Connecticut and New Jersey; the Midwest: Iowa and Michigan; the South: Georgia, Kentucky, and Louisiana; the West: Alaska, California, Hawaii, New Mexico, Utah, and Washington). We excluded patients who were diagnosed before 1990 (N = 155,319), whose gender was male (N = 7077), without information on birth year (N = 77) or accurate follow-up dates after cancer diagnosis (N = 90,462; Online Table 1 presented both baseline and CVD mortality rate), less than 30 years at the time of cancer diagnosis (N = 6159), or whose race was unknown (N = 6043). Finally, we included 924,439 breast cancer patients.

**Table 1.** **Characteristics of breast cancer patients and general female population: a population-based study in the U.S., 1990–2016**.

		Population	Breast cancer patients
		Per 100 PYs (%)	Per 100 PYs (%)
Total person years		7,161,749 (100.00)	67,262 (100.00)
Calendar year at follow-up ^a^			
	1990–1993	901,658 (12.59)	777 (1.15)
	1994–1997	964,870 (13.47)	2646 (3.93)
	1998–2001	1,023,225 (14.29)	5006 (7.44)
	2002–2006	1,349,117 (18.84)	12,819 (19.06)
	2007–2011	1,420,273 (19.83)	20,200 (30.03)
	2012–2016	1,502,607 (20.98)	25,816 (38.38)
Age at follow up, years ^a^			
	30–34	890,584 (12.44)	262 (0.39)
	35–39	899,026 (12.55)	1073 (1.60)
	40–44	888,349 (12.40)	2652 (3.94)
	45–49	839,848 (11.73)	5010 (7.45)
	50–54	764,815 (10.68)	7333 (10.90)
	55–59	659,574 (9.21)	8519 (12.66)
	60–64	555,444 (7.76)	9035 (13.43)
	65–69	469,302 (6.55)	8867 (13.18)
	70–74	389,125 (5.43)	7780 (11.57)
	75–79	319,852 (4.47)	6585 (9.79)
	80–84	242,255 (3.38)	5161 (7.67)
	>85	243,577 (3.40)	4985 (7.41)
Race			
	White	5,699,960 (79.59)	55,014 (81.79)
	Black	769,296 (10.74)	6492 (9.65)
	Other ^b^	692,494 (9.67)	5756 (8.56)
Region of residence ^c^			
	Northeast	980,788 (13.69)	11,710 (17.41)
	Midwest	1,036,734 (14.48)	6479 (9.63)
	South	1,356,826 (18.95)	12,374 (18.40)
	West	3,094,908 (43.21)	36,698 (54.56)
Time since diagnosis to last follow up			
	0 to <1 month	-	755 (1.12)
	1 to <6 month	-	3646 (5.42)
	6 to <12 month	-	4272 (6.35)
	1 to <2 years	-	7760 (11.54)
	2 to <5 years	-	18,656 (27.74)
	5 to <10 years	-	19,302 (28.70)
	≥10 years	-	12,871 (19.14)
Histology			
	Ductal	-	49,220 (73.18)
	Lobular	-	5384 (8.00)
	Mixed	-	6519 (9.69)
	Others	-	6140 (9.13)
Tumor grade			
	Well differentiated	-	12,979 (19.30)
	Moderately differentiated	-	25,838 (38.41)
	Poorly differentiated	-	19,765 (29.38)
	Undifferentiated	-	868 (1.29)
	Unknown	-	7813 (11.62)
Tumor size			
	0–2 cm	-	42,453 (63.11)
	2–5 cm	-	17,963 (26.71)
	>5 cm	-	3413 (5.07)
	Unknown	-	3433 (5.10)
Tumor stage			
	Local	-	45,036 (66.96)
	Regional	-	19,533 (29.04)
	Distant	-	1721 (2.56)
	Unknown	-	971 (1.44)
ER			
	Positive	-	46,896 (69.72)
	Negative	-	11,770 (17.50)
	Unknown	-	8596 (12.78)
PR			
	Positive	-	39,862 (59.26)
	Negative	-	17,496 (26.01)
	Unknown	-	9904 (14.72)
HER2 ^d^			
	Positive	-	1465 (14.20)
	Negative	-	8155 (79.04)
	Unknown	-	697 (6.76)
Molecular subtypes ^d^			
	HR+/HER2+	-	1028 (9.96)
	HR–/HER2+	-	433 (4.20)
	HR+/HER2–	-	7122 (69.03)
	Triple negative	-	1020 (9.88)
	Unknown	-	715 (6.93)
Surgery			
	No/unknown	-	2159 (3.22)
	Yes	-	65,103 (96.79)
Radiotherapy			
	No/unknown	-	33,370 (49.61)
	Yes	-	33,892 (50.39)
Chemotherapy			
	No/unknown	-	41,340 (61.46)
	Yes	-	25,922 (38.54)

Abbreviations: ER, estrogen receptor; PR, progesterone receptor; HR+, hormone-receptor positive; HR–, hormone-receptor negative; HER2, human epidermal growth factor receptor 2; PYs, person-years. ^a^ This was defined as the number of survivors after cancer diagnosis or individuals censused in general population in multiple time periods. ^b^ The other included American Indian/Alaska Native, Asian, and Pacific Islander. ^c^ Breast cancer patients and female population were drawn from SEER which covers 13 states of the total American cancer population. The Northeast included Connecticut and New Jersey. The Midwest included Iowa and Michigan. The South included Georgia, Kentucky, and Louisiana. The West included Alaska, California, Hawaii, New Mexico, Utah, and Washington. Race other than white and black in the population, accounting for 692,494 (9.67%) per 100 person-years, was grouped at the national level given small numbers of cardiovascular deaths. ^d^ Information on HER2 status was available from 2010 onward, and thus the analysis was restricted to patients diagnosed thereafter.

## 3. Ascertainment of Cardiovascular Deaths and Follow-Up Visit

Death certificates dataset was obtained uponalgorithms from tumor sequence, cancer site, and co-existing diseases in SEER database. We utilized the International Classification of Diseases codes (ICD-9, ICD-10) to confirm death from CVD [12]. Cause of death from clinician or coroner coded CVD (ICD-9: 390–448; ICD-10: I00–I78; recode: 50060–50110) was performed including disease of the heart (ICD-9: 390–398, 402, 404, 410–429; ICD-10: I00–I09, I11, I13, I20–I51; recode: 50060), cerebrovascular disease (ICD-9: 430–438; ICD-10: I60–I69; recode: 50080) or other cardiovascular diseases (the remaining codes).

Cancer registration referred to the process of continual, systematic collection of data on the occurrence and characteristics of reportable malignancies. Cancer registrars were responsible for collecting the cancer data and making sure they were timely, accurate, and complete [13]. Follow-up, generated each month with a list of patients due for follow-up compiled and compared to hospital admission and outpatient records, was carried from breast cancer diagnosis to death or December 31, 2016, whichever came first, by linking cancer registries. Attempts were made periodically to contact all patients who do not have a current follow-up.

## 4. Variables

The demographic information included race and living area for breast cancer patients and population, respectively. We obtained age at follow-up and calendar year at follow-up (the number of survivors after cancer diagnosis and individuals censused in general population in multiple time periods) for the two sets and time since diagnosis for patients with breast cancer.

We further derived information on characteristics of tumor and treatments for patients, containing tumor stage, tumor size, histology, tumor grade, the status of estrogen receptor (ER), progesterone receptor (PR), human epidermal growth factor receptor 2 (HER2, accessible after 2010), surgery, radiotherapy, and chemotherapy. Molecular type (accessible after 2010) was divided as hormone receptor-positive (HR+)/HER2–, HR+/HER2+, hormone receptor-negative (HR–)/HER2+, triple-negative, or unknown. The characteristics of cancer survivors and the general female population were shown in Table 1.

## 5. Statistical Analysis

We described the demographic characteristics for breast cancer patients and population, as well as tumor and treatment characteristics for breast cancer patients. Using Poisson regression, we assessed the incidence rate ratios (IRRs) and 95% confidence intervals (95% CIs) of death from CVD in breast cancer patients relative to the general population. In these analyses, we adjusted for age at follow-up, race, region of residence, and calendar year at follow-up.

We calculated the risk of death due to diseases of the heart, cerebrovascular diseases, and other cardiovascular diseases. We estimated IRRs by different time period after cancer diagnosis. We subsequently assessed the subgroup estimates by clinical characteristics of breast cancer patients at the age of follow-up 30–64 years.

STATA (version 14.1; Stata Corporation, College Station, Texas, USA) was used to calculate statistical analyses. *p *< 0.05 shows the significant difference.

## 6. Results

### 6.1 Characteristics of Age at Follow up that Modified the Risk of CVD Mortality 

A total of 924,439 breast cancer patients were diagnosed during 1990–2016. 54,804 CVD deaths (mortality rate: 0.8 per 100 person-years) in breast cancer patients were identified with a median follow-up of 72 months (0.5–323 months). There were 3,431,759 CVD deaths (mortality rate: 0.5 per 100 person-years) in the general female population.

**Fig. 1.**
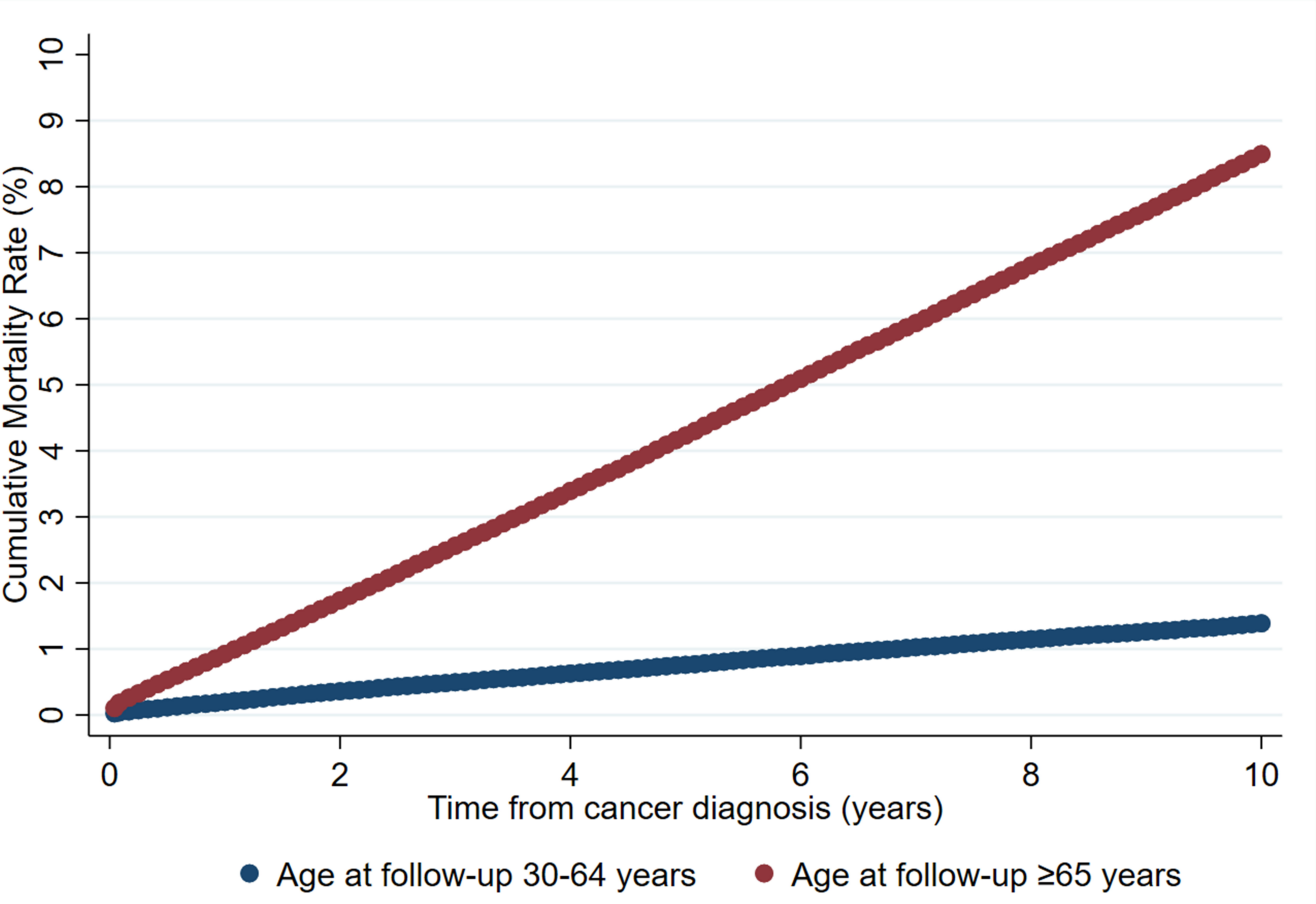
**Cumulative mortality rates of CVD by age at follow up among breast cancer patients from cancer diagnosis to 10 years afterward: a population-based cohort study in the U.S., 1990–2016**.

The cumulative mortality rate of CVD among breast cancer patients at the age of follow up ≥65 years was approximately six times as high as those who were at age of follow up 30–64 years up to 10 years after the cancer diagnosis (8.49% vs. 1.39%; Fig. 1). When compared with the corresponding general population, younger breast cancer patients (age of follow-up 30–64 years) were associated with a mildly increased risk of CVD mortality (IRR 1.06, 95% CI 1.03–1.10) with the adjustment of demographic characteristics. In the subgroup analysis by type-specific CVD, a greater association was found among these patients who died from diseases of the heart (IRR 1.11, 95% CI 1.07–1.15; Table 2). The risk elevation of CVD mortality reached its peak when breast cancer patients were at the age of follow-up 30–34 years (IRR 3.50, 95% CI 1.75–7.01; Table 3).

**Table 2.** ** Incidence rate ratios (IRRs) of type-specific cardiovascular deaths among breast cancer patients, compared with the female population: a population-based study in the U.S., 1990–2016**.

		Population	BCa-1	IRR (95% CI) ^a^
		N (MR)	N (MR)	
Overall cardiovascular deaths				
By age at follow up				
	30–64 years	357,750 (0.07)	3866 (0.11)	1.06 (1.03–1.10)
	>65 years	3,074,009 (1.85)	50,938 (1.53)	0.95 (0.94–0.95)
Disease of the heart				
By age at follow up				
	30–64 years	267,570 (0.05)	3045 (0.09)	1.11 (1.07–1.15)
	>65 years	2,244,200 (1.35)	37,373 (1.12)	0.96 (0.95–0.97)
Cerebrovascular disease				
By age at follow up				
	30–64 years	67,751 (0.01)	576 (0.02)	0.89 (0.82–0.97)
	>65 years	610,310 (0.37)	9832 (0.29)	0.92 (0.90–0.94)
Other cardiovascular diseases				
By age at follow up				
	30–64 years	22,429 (0.00)	245 (0.01)	0.99 (0.87–1.12)
	>65 years	219,499 (0.13)	3733 (0.11)	0.90 (0.87–0.93)

Abbreviations: CI, confidence interval; IRR, incidence-rate ratio; MR, mortality rate per 100 person-years; N, number of deaths. ^a^ IRRs were adjusted for age at follow up (30–34, every five years afterward, or ≥85 years) if applicable, race (white, black, or other), region of residence (Northeast, Midwest, South, or West), and calendar year at follow-up (1990–1993, 1994–1997, 1998–2001, 2002–1006, 2007–2011, or 2012–2016).

**Table 3.** **Incidence rate ratios (IRRs) of cardiovascular mortality in breast cancer patients compared with the general female population: a population-based cohort study in the U.S., 1990–2016**.

		Population	Breast cancer patients	IRR (95% CI) ^a^
		N (MR)	N (MR)	
Overall		3,431,759 (0.48)	54,804 (0.81)	0.95 (0.95–0.96)
By age at follow up, year				
	30–34	7403 (0.01)	8 (0.03)	3.50 (1.75–7.01)
	35–39	13,456 (0.01)	44 (0.04)	2.69 (2.00–3.62)
	40–44	24,355 (0.03)	137 (0.05)	1.91 (1.62–2.26)
	45–49	40,225 (0.05)	310 (0.06)	1.35 (1.20–1.50)
	50–54	61,354 (0.08)	621 (0.08)	1.14 (1.05–1.23)
	55–59	86,454 (0.13)	1034 (0.12)	1.04 (0.98–1.11)
	60–64	124,503 (0.22)	1712 (0.19)	1.00 (0.95–1.05)
	65–69	180,152 (0.38)	2675 (0.30)	0.97 (0.94–1.01)
	70–74	269,853 (0.69)	4266 (0.55)	0.99 (0.96–1.02)
	75–79	407,574 (1.27)	6763 (1.03)	0.98 (0.96–1.01)
	80–84	586,244 (2.42)	10,168 (1.97)	0.97 (0.95–0.99)
	>85	1,630,186 (6.69)	27,066 (5.43)	0.92 (0.91–0.93)
*p* for interaction ^b^				<0.001

Abbreviations: CI, confidence interval; IRR, incidence-rate ratio; MR, mortality rate per 100 person-years; N, number of deaths. ^a^ IRRs were adjusted for age at follow up (30–34, every five years afterward, or ≥85 years), race (white, black, or other), region of residence (Northeast, Midwest, South, or West), and calendar year at follow-up (1990–1993, 1994–1997, 1998–2001, 2002–1006, 2007–2011, or 2012–2016). ^b^ We added an interaction term between breast cancer and age at follow up (30–34, every five years afterward, or ≥85 years) and reported the significance level of the term as *p* for interaction.

### 6.2 Characteristics of Time Since Cancer Diagnosis that Modified the Risk of CVD Mortality

Compared with the population, the risk of CVD mortality among breast cancer patients at the age of follow up 30–64 years was highest within the first month after breast cancer diagnosis (IRR 3.33, 95% CI 2.84–3.91; Table 4) and however showed a decreased, though still elevated, trend after the first month. On the other, breast cancer patients at the age of follow-up ≥65 years were at greatest risk within the first month (IRR 2.19, 95% CI 2.05–2.32) after a cancer diagnosis.

**Table 4.** **Incidence rate ratios (IRRs) of cardiovascular mortality in breast cancer patients by age at follow up and time since cancer diagnosis, compared with the female population: a population-based study in the U.S., 1990–2016**.

		Breast cancer patients	IRR (95% CI) ^a^
		N (MR)	
Age at follow-up 30 to 64 years			
By time since diagnosis			
	0 to <1 month	149 (0.33)	3.33 (2.84–3.91)
	1 to <6 months	278 (0.13)	1.29 (1.15–1.45)
	6 to <12 months	278 (0.11)	1.10 (0.98–1.24)
	1 to <2 years	519 (0.11)	1.15 (1.05–1.25)
	2 to <5 years	1046 (0.10)	0.99 (0.93–1.05)
	5 to <10 years	1047 (0.11)	1.02 (0.96–1.08)
	>10 years	549 (0.12)	0.94 (0.87–1.03)
Age at follow-up ≥65 years			
By time since diagnosis			
	0 to <1 month	1015 (3.40)	2.19 (2.05–2.32)
	1 to <6 months	1884 (1.30)	0.84 (0.81–0.88)
	6 to <12 months	2068 (1.20)	0.78 (0.74–0.81)
	1 to <2 years	4143 (1.28)	0.83 (0.80–0.85)
	2 to <5 years	11,555 (1.38)	0.87 (0.85–0.88)
	5 to <10 years	15,693 (1.58)	0.96 (0.95–0.98)
	>10 years	14,580 (1.75)	1.05 (1.03–1.07)

Abbreviations: CI, confidence interval; IRR, incidence-rate ratio; MR, mortality rate per 100 person-years; N, number of deaths. ^a^ IRRs were adjusted for age at follow up if applicable, race (white, black, or other), region of residence (Northeast, Midwest, South, or West), and calendar year at follow-up (1990–1993, 1994–1997, 1998–2001, 2002–2006, 2007–2011, or 2012–2016).

### 6.3 Clinical Characteristics of Breast Cancer that Modified the Risk of CVD Mortality

Considering that the growth of risk was increased among breast cancer patients at the age of follow-up 30–64 years, we just conducted further analyses in this age group. Associations were stronger in breast cancer patients with a distant stage (IRR 3.46, 95% CI 3.13–3.83), tumor size larger than 5 cm (IRR 2.00, 95% CI 1.82–2.20), poorly/undifferentiated tumor grade (IRRs 1.18–1.22), triple-negative molecular subtype (IRR 1.45, 95% CI 1.18–1.77) or those who did not receive the treatments (e.g., surgery, chemotherapy, radiotherapy) (IRRs 1.09–3.64) (Table 5).

**Table 5.** **Incidence-rate ratios (IRRs) of cardiovascular deaths at the age of follow up 30 to 64 years among breast cancer patients, stratified by clinical characteristics, compared with the female population: a population-based study in the U.S., 1990–2016**.

		Population		Breast cancer patients		IRR (95% CI) ^a^
		100 PYs	N (MR)	100 PYs	N (MR)	
Tumor size						
	0–2 cm	5,497,640	357,750 (0.07)	19,881	1719 (0.09)	0.79 (0.75–0.83) ^c^
	2–5 cm			10,154	1432 (0.14)	1.36 (1.29–1.43) ^c^
	>5 cm			2119	428 (0.20)	2.00 (1.82–2.20) ^c^
	Unknown			1730	287 (0.17)	1.50 (1.33–1.68) ^c^
*p* for difference						<0.001
Tumor stage						
	Local	5,497,640	357,750 (0.07)	20,886	1955 (0.09)	0.85 (0.82–0.89) ^c^
	Regional			11,484	1438 (0.13)	1.22 (1.16–1.29) ^c^
	Distant			1031	375 (0.36)	3.46 (3.13–3.83) ^c^
	Unstaged			482	98 (0.20)	1.91 (1.57-2.33) ^c^
*p* for difference						<0.001
Histology						
	Ductal	5,497,640	357,750 (0.07)	25,540	2,918 (0.11)	1.07 (1.03–1.11) ^c^
	Lobular			2271	215 (0.09)	0.84 (0.73–0.96) ^c^
	Mixed			3231	285 (0.09)	0.85 (0.76–0.96) ^c^
	Others			2841	448 (0.16)	1.41 (1.28–1.54) ^c^
*p* for difference						<0.001
Tumor grade						
	Well differentiated	5,497,640	357,750 (0.07)	5529	530 (0.10)	0.88 (0.81–0.96) ^c^
	Moderately differentiated			12,477	1253 (0.10)	0.95 (0.90–1.01) ^c^
	Poorly differentiated			11,910	1512 (0.13)	1.22 (1.16–1.28) ^c^
	Undifferentiated			517	65 (0.13)	1.18 (0.93–1.51) ^c^
	Unknown			3451	506 (0.15)	1.21 (1.11–1.32) ^c^
*p* for difference						<0.001
Molecular subtypes ^b^						
	HR+/HER2+	5,497,640	357,750 (0.07)	689	56 (0.08)	0.95 (0.73–1.23) ^c^
	HR–/HER2+			294	29 (0.10)	1.09 (0.76–1.57) ^c^
	HR+/HER2–			3738	324 (0.09)	0.95 (0.85–1.06) ^c^
	Triple negative			648	94 (0.15)	1.45 (1.18–1.77) ^c^
	Unknown			378	65 (0.17)	1.05 (1.01–1.10) ^c^
*p* for difference						0.011
Chemotherapy						
	No/unknown	5,497,640	357,750 (0.07)	15,620	1995 (0.13)	1.09 (1.04–1.14) ^c^
	Yes			18,263	1871 (0.10)	1.04 (1.00–1.09) ^c^
*p* for difference						0.111
Radiotherapy						
	No/unknown	5,497,640	357,750 (0.07)	16,308	2310 (0.14)	1.33 (1.27–1.38) ^c^
	Yes			17,575	1556 (0.09)	0.82 (0.78–0.86) ^c^
*p* for difference						<0.001
Surgery						
	No/unknown	5,497,640	357,750 (0.07)	1028	417 (0.41)	3.64 (3.31–4.01) ^c^
	Yes			32,814	3440 (0.10)	0.98 (0.95–1.01) ^c^
*p* for difference						<0.001

Abbreviations: CI, confidence interval; IRR, incidence-rate ratio; MR, mortality rate per 100 person-years; HR+, hormone-receptor positive; HR–, hormone-receptor negative; HER2, human epidermal growth factor receptor 2; PYs, person-years; N, number of death. ^a^ IRRs were adjusted for age at follow up (30–34, every five years afterward, or 60–64 years) race (white, black, or other), region of residence (Northeast, Midwest, South, or West), and calendar year at follow-up (1990–1993, 1994–1997, 1998–2001, 2002–2006, 2007–2011, or 2012–2016). ^b^ Information on HER2 status was available from 2010 onward, and thus the analysis was restricted to patients diagnosed thereafter. ^c^ These IRRs were estimated by comparing different tumor and treatment characteristics of breast cancer patients with the general population. We tested the difference of IRRs across characteristics using Wald test and reported the *p* value for significance.

## 7. Discussion

The aim of this study is to address a knowledge gap in the relative risk of CVD mortality among breast cancer patients by age at follow-up and time since diagnosis, suggesting age-specific and time-dependent disease course. Our findings reveal the risk of CVD mortality among breast cancer patients is lower than that in the general population but increased in patients at the age of follow-up 30–64 years. The elevated risk was highest among patients within the first month after cancer diagnosis and at age of follow up 30–34 years. Stronger relationships were also found for younger patients with aggressive tumor characteristics or those who did not receive the treatments.

Age, one of the most critical risk factors shared by CVD and breast cancer, is positively associated with an absolute increased risk of CVD mortality [5, 14]. A study suggested an elevated risk of CVD mortality was found (standardized mortality rate [SMR] 1.38, 95% CI 1.00–1.84) among breast cancer patients at the age of follow-up 55–64 years, compared with general population [15]. Our results further suggested an increased risk of CVD mortality in breast cancer patients at the age of follow-up 30–64 years, but a mildly decreased risk was observed among those ≥65 years. A previous study reported an increase in CVD-related death among 1413 breast cancer survivors compared to age-matched women without breast cancer was observed only seven years after diagnosis (IRR 1.8, 95% CI 1.3–2.5) [7]. Our findings indicated that the risk of CVD mortality was greater among younger patients during the whole follow-up period relative to the general population. The conclusion was consistent with the result from a recent study that the younger a cancer survivor was diagnosed, the higher the relative risk would be [3]. A recent study suggested that compared to age-matched women without breast cancer, breast cancer survivors aged 70–79 at diagnosis of localized breast cancer had a lower multivariate-adjusted risk of CVD mortality (IRR 0.84, 95% CI 0.70–1.00) [10]. Our data extensively revealed the decreased risk among younger breast cancer survivors with a localized tumor stage, implying that early screening and diagnosis of breast cancer in clinical practice may reduce the risk of CVD mortality.

Irradiation therapy may increase the risk of CVD mortality by activating acute inflammatory cascades and develop myocardial fibrosis leading to the injury of cardiac muscle or the surrounding vasculature [16, 17, 18]. However, our findings, in contrast to some others [19, 20], of reduced risk in CVD mortality among breast cancer patients receiving radiotherapy suggested that irradiation was less hazardous to the heart and more targeted to breast cancer after the 1990s with the development and improvement of techniques and regimens. In addition, some chemotherapy (e.g., anthracycline, trastuzumab) may lead to an increased risk of CVD mortality by damaging the circulatory system [5, 21]. Our results of a higher risk of CVD mortality among younger breast cancer patients who received chemotherapy validated this statement. Moreover, the stratification analysis by cancer stage suggested the higher cancer stage was, the more risky in terms of CVD mortality, which was supported by previous studies [22], indicating the importance of long term concern for CVD among younger patients with breast cancer who received chemotherapy (especially anthracycline and trastuzumab). Besides, the IRRs showed a greater risk of CVD mortality for breast cancer patients at the age of follow-up 30 to 64 years who didn’t receive chemotherapy, which should be concerned given that these patients were different from those who received chemotherapy regarding their baseline (e.g., older age at follow up. Online Table 2), suggesting they had more comorbidities.

A large number of studies have investigated the elevated risk of CVD among adults experiencing psychological stress during the past decades. Psychological stress caused to acute impairment of endothelial function, elevation of inflammatory cytokines (e.g., interleukin-6 and tumor necrosis factor-α in circulation), platelet activation, prothrombotic changes in molecules involved in coagulation [23] and activation of sympathetic nervous system [24, 25]. Previous studies explored the transiently increased risks of CVD mortality after the diagnosis of cancer [8, 26]. Resemblance to these results, we also found the immediately increased risk of CVD mortality within the first month after breast cancer diagnosis relative to the general population. Therefore, psychosocial factors should be assessed by clinical interview, and tailored clinical management coupled with education should be recommended for those at high-stress risk [23].

This is a population-based prospective cohort study with the mitigation of recall biases, which lends support to illuminate associations with clinical characteristics, age at follow-up, and time since diagnosis. However, there are some limitations in this study. First, data on the general female population is not cancer-free leading to underestimation of the true risk. Second, although we carefully adjusted for age at follow up, calendar time, race, and region of residence in the IRRs calculation, other potential confounding factors (such as physical or mental health status or presence of comorbidities like body mass index, hypertension, diabetes) which may be related to CVD death could not be addressed. However, the fact that the increased risk of cardiovascular deaths was manifest in the first month after cancer diagnosis alleviates this concern. Moreover, patients with no accurate follow-up dates after breast cancer diagnosis were eliminated. Nevertheless, the CVD mortality rate was much higher in the exclusive patients than the inclusive ones (1.08 vs. 0.81 per 100 person-years), leading to the underestimated relationship. In addition, although the distribution of age at follow-up was uneven between breast cancer patients and general population, we explored the association by age at follow-up groups (Table 3) where subjects between the two groups were analyzable. Last, given the short follow up time, future study with a longer follow up is needed.

## 8. Conclusions

It is important to identify breast cancer patients at increased risk of CVD mortality and health facilities should provide risk mitigation strategies with early monitoring for breast cancer survivors especially those who were young or with aggressive tumor stage. Psychosocial factors should be assessed by clinical interview simultaneously with the diagnosis of breast cancer.

## Data Availability

The data is publicly available from the SEER Program (https://seer.cancer.gov/).
